# Risk factors of radiographic severity of massive rotator cuff tear

**DOI:** 10.1038/s41598-022-17624-y

**Published:** 2022-08-09

**Authors:** Ryogo Furuhata, Noboru Matsumura, Satoshi Oki, Takahiro Nishikawa, Hiroo Kimura, Taku Suzuki, Masaya Nakamura, Takuji Iwamoto

**Affiliations:** 1 Department of Orthopaedic Surgery, Keio University School of Medicine, 35 Shinanomachi, Shinjuku-ku, Tokyo 160-8582 Japan; 2 Department of Orthopaedic Surgery, Saiseikai Utsunomiya Hospital, Utsunomiya-shi, Tochigi Japan

**Keywords:** Trauma, Risk factors, Musculoskeletal system

## Abstract

As massive rotator cuff tears progress, various radiographic changes occur; however, the factors associated with radiographic changes remain largely unknown. This study aimed to determine the factors that affect radiographic severity in massive rotator cuff tears using multivariate analyses. We retrospectively reviewed 210 shoulders with chronic massive rotator cuff tears. The dependent variables were superior migration of the humeral head (Hamada grades 2–3), narrowing of the glenohumeral joint (grade 4), and humeral head collapse (grade 5). Baseline variables that were significant in univariate analyses were included in multivariate models. There were 91, 59, 43, and 17 shoulders classified as Hamada grades 1, 2–3, 4, and 5, respectively. Multivariate analysis showed that infraspinatus tear (*P* = 0.015) and long head of biceps (LHB) tendon rupture (*P* = 0.007) were associated with superior migration of humeral head. Superior subscapularis tear (*P* = 0.003) and LHB tendon rupture (*P* < 0.001) were associated with narrowing of glenohumeral joint. Female sex (*P* = 0.006) and superior subscapularis tear (*P* = 0.006) were associated with humeral head collapse. This study identified the rupture of infraspinatus and LHB as risk factors of superior migration of humeral head, and the rupture of subscapularis and LHB and female sex as risk factors of cuff tear arthropathy.

## Introduction

**Figure 1:**
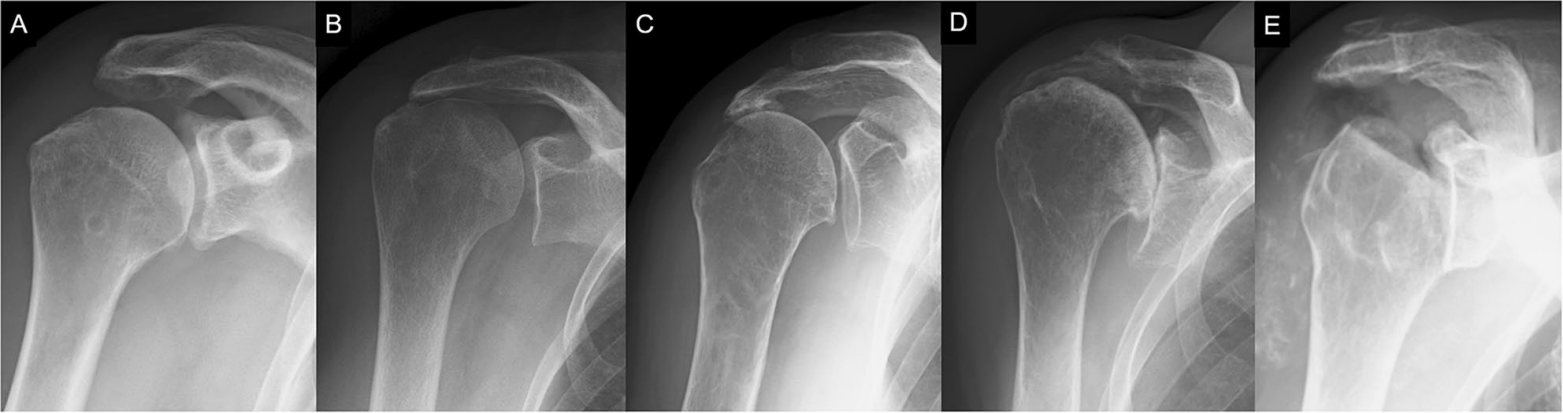
Radiographic images showing different grades of the Hamada classification of massive rotator cuff tears. **(A)** Grade 1 is characterized by a maintained acromiohumeral interval. **(B)** Grade 2 shows narrowing of the acromiohumeral interval. **(C)** Grade 3 shows subacromial acetabulization in addition to grade 2 features. **(D)** Grade 4 shows narrowing of the glenohumeral joint in addition to grade 3 features. **(E)** Grade 5 indicates humeral head collapse.

As massive rotator cuff tears progress, radiographic changes such as superior migration of the humeral head, osteoarthritis of the glenohumeral joint, and humeral head collapse are observed^1–7^. Hamada classification is widely used for radiographic classification of massive rotator cuff tears that reflect these changes^4,5^. This classification assigns massive rotator cuff tears to five radiographic grades: preserved acromiohumeral interval (grade 1); narrowing of acromiohumeral interval (grade 2); subacromial acetabulization in addition to grade 2 features (grade 3); narrowing of the glenohumeral joint in addition to grade 3 features (grade 4); and collapse of the humeral head (grade 5) (Fig. 1)^4,5^. Although Hamada classification remains unclear whether it progresses according to the grade, clinically this radiographic classification often has a great influence on the clinical outcome and treatment strategy. As a clinical outcome, a correlation between Hamada classification grades and Constant–Murley scores^8^ has been reported^9^. Hamada grades 2 and 3, which are cases with superior migration of the humeral head, present with retears more frequently than grade 1 following rotator cuff repair^5,10,11^. Cuff tear arthropathy, which corresponds to Hamada grades 4 and 5, is generally indicated in shoulder arthroplasty^1,3,12^. To predict patients’ prognosis, it is important to identify the radiographic severity factors associated with massive rotator cuff tears.

However, the mechanism of radiographic progression of massive rotator cuff tears has not been fully elucidated, and few studies^5,13^ examined the risk factors of progression. Hamada et al.^5^ reported that Hamada grades 3–5 have a significantly higher proportion of subscapularis (SSC) and teres minor (TM) tears than grade 1, which suggests that the status of SSC and TM contributes to the progression of massive rotator cuff tears. However, Walch et al.^13^ identified age at the time of surgery, delay of surgery, duration of follow-up, status of TM, and fatty infiltration of the infraspinatus (ISP) and SSC as factors that affected the progression of Hamada arthritis stage in patients who underwent only arthroscopic tenotomy of the long head of biceps (LHB) for rotator cuff tears. Although these recent advances have led to a better understanding of the pathogenesis of cuff tear arthropathy, multiple factors are considered to affect the progression to a higher grade in the Hamada classification system, and these factors have not been fully elucidated. This study aimed to use multivariate analyses to identify the predictive factors affecting radiographic severity in massive rotator cuff tears.

## Materials and methods

This study was approved by the Institutional Review Board of the Keio University School of Medicine (Reference study number: 20130147). All methods were conducted in accordance with relevant guidelines and regulations. Opt-out consent method was performed for each patient on our hospital’s bulletin board and web site. Opt-out consent relies on implicit consent, where willingness to participate is tacit or presumed and can be retracted by active objection.

### Patient selection

The present study was a retrospective study and involved patients who presented with massive rotator cuff tears between April 2011 and April 2021. Based on previous reports^14^, we defined massive rotator cuff tears as a complete tear of two or more tendons in the rotator cuff as diagnosed by magnetic resonance imaging (MRI). The inclusion criterion was a chronic massive rotator cuff tear of more than six months after the onset of symptoms. In addition, patients whose plain radiography and MRI were performed within three months from the evaluation of physical findings and medical history was obtained at the initial visit were included in the study. The exclusion criteria were patients with limited passive range of shoulder motion, septic shoulder arthritis, or previous shoulder surgery.

In this study, we identified 210 shoulders that fulfilled the inclusion criteria. The mean age of the patients was 72.9 ± 8.3 years (range: 45–89 years). The mean time from symptom onset was 2.9 ± 3.7 years (range: 0.5–20 years). Eighty shoulders (38.1%) had a history of trauma.

### Outcome measures

We classified the patients who fulfilled the eligibility criterion as having Hamada classification^4,5^ grade 1, 2, 3, 4, or 5 based on plain radiographic findings. One examiner evaluated the involved tendon using MRI, with the results of plain radiographic findings blinded. Plain radiographs were taken with the patient in a standing position, and MRI was performed in the supine position. In SSC tears, the upper 2/3 of the SSC attaches to the lesser tuberosity as a tendon, while the lower 1/3 attaches to the muscle^15,16^. Therefore, superior SSC and inferior SSC are considered to have different functions^17,18^; we evaluated SSC tears separately for superior and inferior SSC in this study. In addition, since the inferior SSC and TM attach to the humerus as muscles and not tendons^15,16,19^, it is difficult to determine the presence of tears; therefore, we defined inferior SSC tears or TM tears as fatty infiltration into the muscles as Goutallier classification^20^ grade 3 or higher^17^.

We investigated the risk factors of migration of the humeral head, narrowing of the glenohumeral joint, and humeral head collapse in patients with massive rotator cuff tears. The dependent variables were Hamada grades 2–3 for superior migration of the humeral head in shoulders without glenohumeral arthritis (Hamada grades 1–3), Hamada grade 4 for narrowing of the glenohumeral joint in shoulders without humeral head collapse (Hamada grades 1–4), and Hamada grade 5 for humeral head collapse. The Hamada classification is the radiographic grading of massive rotator cuff tear, but it remains unclear whether it progresses accordingly. However, it is unlikely to drop from a higher grade to a lower grade; therefore, we compared Hamada grade 1 versus grades 2–3, grades 1–3 versus grade 4, and grades 1–4 versus grade 5 in this study. Explanatory variables included age, sex, duration of symptoms, trauma, smoking, diabetes, hypertension, rheumatoid arthritis, pseudoparalysis, ISP tear, superior SSC tear, fatty infiltration into supraspinatus (SSP), ISP, superior SSC, inferior SSC, and TM, and LHB tendon rupture. Medical history was evaluated based on the clinical notes. Based on the previous definition of pseudoparalysis^21^, we defined the active elevation of the shoulder that is limited to 90° as pseudoparalysis in this study. Two orthopedic surgeons with over 10 years of experience in shoulder surgery assessed the range of shoulder motion using a goniometer. In addition, we evaluated fatty infiltration into each rotator cuff muscle with T1 sagittal oblique MRI immediately lateral to the scapular spine’s attachment to the body of the scapula and defined the presense of fatty infiltration as Goutallier grade 3 or higher, because a previous report^22^ has shown a significant association between fatty infiltration into these muscles of Goutallier's grade 3 or higher and superior migration of humeral head.

### Statistical analysis

All statistical analyses were conducted using the SPSS software (Version 26.0, IBM Corp., Armonk, NY, USA). In univariate analysis, we used Student's t-tests to compare the average of continuous values, such as age and duration of symptoms. In contrast, chi-squared tests were used to compare the proportion of discrete variables, such as sex, history of trauma, smoking, medical history of diabetes, hypertension, rheumatoid arthritis, involved tendons, and LHB tendon rupture. Baseline variables, which were statistically significant in the univariate analysis, were included in the multivariable models. Multivariate analyses were performed using logistic regression analysis to identify the independent predictors of superior migration of the humeral head, narrowing of the glenohumeral joint, and humeral head collapse. The Cox and Snell R square and the Nagelkerke R square values were calculated to evaluate the variability explained by the regression model. Regression model fit was estimated using the Hosmer–Lemeshow goodness-of-fit test. Accuracy of this model was calculated as follows; (true positive) + (true negative)/(true positive) + (false positive) + (true negative) + (false negative). Furthermore, we developed a logistic regression equation for independent predictors derived from logistic regression analyses. We create the receiver operating characteristic (ROC) curve of the regression equation and calculated the area under the curve (AUC) to evaluate the equation’s predictive value. In this study, we compared Hamada grade 1 versus grades 2–3, grades 1–3 versus grade 4, and grades 1–4 versus grade 5 to clarify the association with each radiographic findings; however, repeating statistical tests in the same population may have created an ɑ error. Therefore, the statistical significance level was set at 0.017, divided by 3, rather than 0.05, based on the Bonferroni’s correction.

## Results

**Figure 2:**
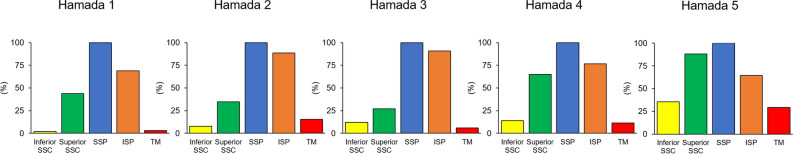
The graphs indicate the proportion of the involved tendon of the rotator cuff in Hamada grades 1, 2,3, 4, and 5. *SSC* subscapularis, *SSP* supraspinatus, *ISP* infraspinatus, *TM* teres minor.

Hamada grade 1 included 91 shoulders (43.3%), grade 2 included 26 (12.4%), grade 3 included 33 (15.7%), grade 4 included 43 (20.5%), and grade 5 included 17 (8.1%). SSP tears were observed in 210 shoulders: ISP tears in 160 (76.2%), superior SSC tears in 100 (47.6%), inferior SSC tears in 21 (10.0%), and TM tears in 17 (8.1%). Figure 2 shows the proportion of involved tendons according to the Hamada classification. Hamada grade 2 or 3 had a higher rate of ISP tear (88.5% and 90.9%, respectively) than grade 1 or 5 (68.8% and 64.7%, respectively). As the radiographic grade progressed to Hamada 4 or 5, the rate of superior SSC tears increased (65.1% and 88.2%, respectively). In addition, Hamada grade 5 had a higher proportion of inferior SSC and TM tears than other grades (35.3% and 29.4%, respectively).

### Hamada grade 1 versus 2–3 (superior migration of humeral head)

In the univariate analyses, superior migration of the humeral head was significantly associated with ISP tear (*P* = 0.005), fatty infiltration into SSP (*P* = 0.006), and LHB tendon rupture (*P* = 0.003).

Multivariate analysis showed that risk factors of superior migration of the humeral head were ISP tear (odds ratio 3.51, 95% CI 1.28–9.62; *P* = 0.015) and LHB tendon rupture (odds ratio 3.74; 95% CI 1.43–9.80; *P* = 0.007). The Cox and Snell R square and the Nagelkerke R square values were 0.139 and 0.189, respectively. The Hosmer–Lemeshow goodness-of-fit test showed no significant difference from good model fit (*P* = 0.906). Accuracy of this model was 67.3%. The AUC of ROC curve was 0.715 (95% CI 0.633–0.798), implying that the equation may be used to classify 71.5% of superior migration of humeral head (Table 1). The predicted probability based on four possible combinations of the two independent predictors was 70.7% (ISP tear: YES, LHB tendon rupture: YES), 30.4% (YES, NO), 31.6% (NO, YES), and 11.0% (NO, NO), respectively.

**Table 1:** Univariate and multivariate predictors of superior migration of the humeral head.

Variables	Univariate predictors			Multivariate predictors	
	Hamada grade 1 (N = 91)	Hamada grades 2 and 3 (N = 59)	*P*-value	Odds ratio (95% CI)	*P*-value
Age (years)	72.2 ± 8.2	71.8 ± 9.1	0.810	–	–
Sex (female)	46 (51%)	29 (49%)	0.867	–	–
Duration of symptoms (years)	2.9 ± 3.7	2.6 ± 2.8	0.441	–	–
Trauma	33 (36%)	29 (49%)	0.117	–	–
Smoking	31 (34%)	17 (29%)	0.501	–	–
Diabetes	20 (22%)	10 (17%)	0.452	–	–
Hypertension	37 (41%)	23 (39%)	0.838	–	–
RA	7 (8%)	3 (5%)	0.532	–	–
Pseudoparalysis	22 (24%)	22 (37%)	0.085		
ISP tear	64 (70%)	53 (90%)	0.005*	3.51 (1.28–9.62)	0.015*
Superior SSC tear	39 (43%)	18 (31%)	0.128	–	–
FI into SSP	33 (36%)	35 (59%)	0.006*	2.34 (1.15–4.75)	0.019
FI into ISP	17 (19%)	17 (29%)	0.148	–	–
FI into superior SSC	29 (32%)	13 (22%)	0.190	–	–
FI into inferior SSC	2 (2%)	7 (12%)	0.029	–	–
FI into TM	3 (3%)	6 (10%)	0.083	–	–
LHB tendon rupture	8 (9%)	16 (27%)	0.003*	3.74 (1.43–9.80)	0.007*

Continuous data are presented as mean ± standard deviation.

*CI* Confidence interval, *RA* Rheumatoid arthritis, *ISP* Infraspinatus, *SSC* Subscapularis, *FI* Fatty infiltration of Goutallier grade 3 or higher, *TM* Teres minor, *LHB* Long head of biceps brachii.

*P < 0.017.

### Hamada grade 1–3 versus 4 (narrowing of glenohumeral joint)

In the univariate analyses, narrowing of the glenohumeral joint was significantly associated with superior SSC tear (*P* = 0.002), and LHB tendon rupture (*P* < 0.001).

Multivariate analysis showed that risk factors of narrowing of the glenohumeral joint were superior SSC tear (odds ratio 3.23; 95% CI 1.50–6.95; *P* = 0.003) and LHB tendon rupture (odds ratio 6.31; 95% CI 2.98–13.68; *P* < 0.001). The Cox and Snell R square and the Nagelkerke R square values were 0.155 and 0.237, respectively. The Hosmer–Lemeshow goodness-of-fit test showed no significant difference from the good model fit (*P* = 0.445). Accuracy of this model was 79.3%. The AUC of ROC curve was 0.767 (95% CI 0.688–0.847), implying that the equation may be used to classify 76.7% of narrowing of glenohumeral joint (Table 2). The predicted probability based on four possible combinations of the two independent predictors was 63.4% (Superior SSC tear: YES, LHB tendon rupture: YES), 21.6% (YES, NO), 35.0% (NO, YES), and 7.9% (NO, NO), respectively.

**Table 2:** Univariate and multivariate predictors of osteoarthritis of glenohumeral joint.

Variables	Univariate predictors			Multivariate predictors	
	Hamada grades 1–3 (N = 150)	Hamada grade 4 (N = 43)	*P*-value	Odds ratio (95% CI)	*P*-value
Age (years)	72.0 ± 8.6	75.1 ± 6.9	0.020	–	–
Sex (female)	75 (50%)	25 (58%)	0.346	–	–
Duration of symptoms (years)	2.8 ± 3.5	2.9 ± 3.0	0.767	–	–
Trauma	62 (41%)	12 (28%)	0.110	–	–
Smoking	48 (32%)	15 (35%)	0.722	–	–
Diabetes	30 (20%)	11 (26%)	0.430	–	–
Hypertension	60 (40%)	20 (47%)	0.445	–	–
RA	10 (7%)	1 (3%)	0.279	–	–
Pseudoparalysis	44 (23%)	19 (44%)	0.067		
ISP tear	117 (78%)	33 (77%)	0.862	–	–
Superior SSC tear	57 (38%)	28 (65%)	0.002*	3.23 (1.50–6.95)	0.003*
FI into SSP	68 (45%)	25 (58%)	0.138	–	–
FI into ISP	34 (23%)	14 (33%)	0.186	–	–
FI into Superior SSC	42 (28%)	18 (42%)	0.083	–	–
FI into inferior SSC	8 (5%)	6 (14%)	0.055	–	–
FI into TM	9 (6%)	5 (12%)	0.210	–	–
LHB tendon rupture	24 (16%)	23 (53%)	< 0.001*	6.31 (2.91–13.68)	< 0.001*

Continuous data are presented as mean ± standard deviation.

*CI* Confidence interval, *RA* Rheumatoid arthritis, *ISP* Infraspinatus, *SSC* Subscapularis, *FI* Fatty infiltration of Goutallier grade 3 or higher, *TM* Teres minor, *LHB* Long head of biceps brachii.

*P < 0.017.

### Hamada grades 1–4 versus 5 (humeral head collapse)

In the univariate analyses, the collapse of the humeral head was significantly associated with female sex (*P* = 0.004), pseudoparalysis (*P* < 0.001), superior SSC tear (*P* = 0.001), infiltration into superior SSC (*P* = 0.005), fatty infiltration into inferior SSC (*P* < 0.001), and fatty infiltration into TM (*P* = 0.002).

Multivariate analysis showed that risk factors of collapse of the humeral head were female sex (odds ratio 10.30, 95% CI 1.98–54.43; *P* = 0.006), superior SSC tear (odds ratio 15.81, 95% CI 2.17–115.00; *P* = 0.006), and fatty infiltration into inferior SSC tear (odds ratio 5.57, 95% CI 1.24–25.10; *P* = 0.025). The Cox and Snell R square and the Nagelkerke R square values were 0.175 and 0.406, respectively. The Hosmer–Lemeshow goodness-of-fit test showed no significant difference from the good model fit (*P* = 0.962). Accuracy of this model was 91.9%. The AUC of ROC curve was 0.838 (95% CI 0.739–0.936), implying that the equation may be used to classify 83.8% of humeral head collapse (Table 3). The predicted probability based on four possible combinations of the two independent predictors was 20.8% (Female: YES, Superior SSC tear: YES), 1.6% (YES, NO), 2.5% (NO, YES), and 0.2% (NO, NO), respectively.

**Table 3:** Univariate and multivariate predictors of humeral head collapse.

Variables	Univariate predictors			Multivariate predictors	
	Hamada grades 1–4 (N = 193)	Hamada grade 5 (N = 17)	*P*-value	Odds ratio (95% CI)	*P*-value
Age (years)	72.7 ± 8.3	75.4 ± 7.2	0.169	–	–
Sex (female)	100 (52%)	15 (88%)	0.004*	10.30 (1.98–54.43)	0.006*
Duration of symptoms (years)	2.8 ± 3.4	5.2 ± 6.3	0.396	–	–
Trauma	74 (38%)	6 (35%)	0.804	–	–
Smoking	63 (33%)	4 (24%)	0.440	–	–
Diabetes	41 (21%)	1 (6%)	0.129	–	–
Hypertension	80 (41%)	8 (47%)	0.653	–	–
RA	11 (6%)	1 (6%)	0.975	–	–
Pseudoparalysis	63 (33%)	13 (76%)	< 0.001*	3.99 (0.99–16.17)	0.053
ISP tear	150 (78%)	11 (65%)	0.224	–	–
Superior SSC tear	85 (44%)	15 (88%)	0.001*	15.81 (2.17–115.00)	0.006*
FI into SSP	93 (48%)	12 (76%)	0.076	–	–
FI into ISP	48 (25%)	6 (35%)	0.346	–	–
FI into superior SSC	60 (31%)	11 (65%)	0.005*	0.35 (0.07–1.83)	0.214
FI into inferior SSC	15 (8%)	7 (41%)	< 0.001*	5.57 (1.24–25.10)	0.025
FI into TM	14 (7%)	5 (29%)	0.002*	4.65 (0.84–25.62)	0.078
LHB tendon rupture	46 (24%)	7 (41%)	0.115	–	–

Continuous data are presented as mean ± standard deviation.

*CI* Confidence interval, *RA* Rheumatoid arthritis, *ISP* Infraspinatus, *SSC* Subscapularis, *FI* Fatty infiltration of Goutallier grade 3 or higher, *TM* Teres minor, *LHB* Long head of biceps brachii.

*P < 0.017.

## Discussion

In this study, we examined the relationship between radiographic findings and ruptured tendons in a massive rotator cuff tear. Furthermore, we conducted multivariate analyses to identify the predictive factors that affect the presence of superior migration of the humeral head, narrowing of the glenohumeral joint, and collapse of the humeral head in massive rotator cuff tears. As a result, this study showed that as the radiographic grade progressed to Hamada grades 4–5, the ratio of SSC and TM tears increased, whereas the ratio of ISP tears was high in grades 2–3. In addition, we identified ISP tear and LHB tendon rupture as risk factors of superior migration of the humeral head, superior SSC tear and LHB tendon rupture as risk factors of narrowing of the glenohumeral joint, and female sex and superior and inferior SSC tears as risk factors of humeral head collapse.

Superior migration of the humeral head, which is a characteristic finding of massive rotator cuff tears, is assumed to occur by the breakdown of the force couples in the coronal plane, consisting of the superiorly directed force vector of the deltoid and the inferiorly directed force vector of the rotator cuff muscle^6^. Specifically, since ISP plays a major role in regulating the humeral head inferiorly when the SSP is torn, superior migration of the humeral head reflects the presence of rotator cuff tears, especially multiple-tendon rotator cuff tears involving the ISP^18,23^. This study indicated that ISP tears, in addition to SSP tears, are a significant risk factor of superior migration of the humeral head; our results were consistent with those in the past reports^18,23^. In another clinical study, as fatty infiltration of SSP and ISP progressed, the ratio of patients with superior migration of the humeral head increased^22^, suggesting that the degeneration of the SSP and ISP is associated with the onset of superior migration of the humeral head. However, in this study, fatty infiltration of SSP was significantly associated with superior migration of the humeral head only in univariate analysis, but not in multivariate analysis, which suggests that the presence of ISP tendon tear may be more predictive for superior migration of the humeral head than the change in volume of fatty infiltration of SSP or ISP. In addition, this study suggested a significant association between LHB tendon rupture and superior migration of the humeral head; however, there has been some debate as to whether LHB tendon rupture is a cause or a consequence of superior migration of the humeral head^4,5,13,22,24–26^. LHB tendon acts as a humeral head depressor and shoulder stabilizer, and its rupture causes superior migration of the humeral head^4,24–26^. Hamada et al.^5^ reported that LHB tendon rupture was significantly more common in Hamada grades 3–5 cases than in cases of grade 1 or 2, which is consistent with the results of the present study. However, arthroscopic biceps tenotomy without rotator cuff repair was reported to have no mid- to long-term influence on progressive radiographic changes^13^, and LHB tendon rupture in patients with rotator cuff tears did not significantly reduce the acromiohumeral interval^22^. These studies^13,22^ raised the possibility that LHB tendon rupture was not the specific cause of superior migration of the humeral head, but the result of grade progression. Although this study was able to demonstrate an association between LHB tendon rupture and superior migration of the humeral head, the causal association remains unclear.

In the mechanism of osteoarthritis of the glenohumeral joint in patients with massive rotator cuff tears, imbalance in the strength of the internal and external rotators associated with massive rotator cuff tears is considered to cause transverse and axial instability in the glenohumeral joint, resulting in narrowing of the glenohumeral joint and anterior subluxation and medialization of the humeral head^4^. The SSC is known to form the anterior portion of the transverse force couple, contributing to dynamic glenohumeral stability^18,27–29^. Specifically, the intra-articular component of the superior SSC tendon acts as the primary restraint to anterior glenohumeral translation in the glenohumeral motion from neutral to midrange^30^. Therefore, disruption of the SSC is assumed to cause muscular imbalance and subsequent glenohumeral microinstability^31,32^. In a recent clinical study, SSC repair failure was reported to significantly increase the risk of developing secondary glenohumeral osteoarthritis^33^, indicating that dysfunction of the SSC contributes to the progression of glenohumeral osteoarthritis. These findings support the view that the tear of the superior SSC caused glenohumeral anterior micro-instability, leading to the onset of cuff tear arthropathy. Furthermore, because biomechanical studies have shown that LHB also contributes to glenohumeral joint stability in all directions in addition to the stability in the coronal plane^34–36^, glenohumeral micro-instability associated with LHB tendon rupture might explain the association between LHB tendon rupture and glenohumeral osteoarthritis in this study.

Humeral head collapse is considered an end-stage change in plain radiographs associated with massive rotator cuff tears. Neer et al.^7^ proposed mechanical and nutritional factors for the onset of humeral head collapse. Mechanical factors include anteroposterior instability of the glenohumeral joint caused by a rotator cuff tear^7^. Neer et al.^7^ reported that repetitive trauma from the altered biomechanics associated with glenohumeral joint stabilizers causes glenohumeral articular wear. Nutritional factors include leakage of synovial fluid, which reduces the perfusion of nutrients into the articular cartilage by a loss of closed joint space, and joint inactivity, leading to structural alteration in articular cartilage^7^. These factors cause histological atrophy of the articular cartilage and osteoporosis of the subchondral bone of the humeral head, ultimately leading to humeral head collapse^7^. In this study, superior SSC tear contributed to the onset of humeral head collapse. Inferior SSC status was also strongly associated with humeral head collapse, although not statistically significant. Inferior SSC has been reported to contribute to the stability of the glenohumeral joint for anterior translation by positioning anterosuperior to the humeral head in mid-range abduction^37^. In a cadaveric study^18^, the radius of the trajectory of the glenohumeral joint was significantly greater only in the model without traction force on SSP and superior and inferior SSC compared to the model with static loading on all rotator cuff muscles. Therefore, if dysfunction of inferior SSC occur in addition to superior SSC tears, further anteroposterior instability develops in the glenohumeral joint, thereby increasing the risk for humeral head collapse. In addition, the anterior humeral circumflex vessels, which provide the blood supply to the humeral head^38,39^, run through the dorsal margin of the SSC^40^. Thus, vessel rupture with SSC tear may be another cause of humeral head collapse. TM also increases the compression force across the glenohumeral joint in end-range motion and stabilizes the glenohumeral joint especially in the anterior direction^41^; however, fatty infiltration into TM was significantly associated with humeral head collapse only in univariate analysis and not in multivariate analysis. These results suggest that SSC has more influence on humeral head collapse than TM. The mechanism of association between female sex and humeral head collapse has not been clarified; however, the lower bone mineral density of the proximal humerus in women^42^ may contribute to the onset of subchondral collapse. Pseudoparalysis was significantly associated with humeral head collapse in univariate analysis, but not in multivariate analysis. This discrepancy may be explained by the possible confounding relationship between the status of inferior SSC and pseudoparalysis, as these factors have been shown to be associated^17,43^.

This study has several major limitations. First, explanatory variables in this study do not fully explain the objective variable and there are factors not measured in this study that may be residual confounding, because R square values in the logistic regression analysis of this study are small. For example, patients’ activity, deltoid function, and strength of each patient’s bone might affect the severity of radiographic findings. Second, patients more often presented to our institution for the purpose of surgery because the institutions participating in this study were general hospitals where surgery could be performed. Few patients with asymptomatic massive rotator cuff tears were included in this study. This may have caused a selection bias in this study. Third, ruptures of inferior subscapularis and teres minor in this study were determined from fatty infiltration into these muscles on MRI, which may not accurately reflect rupture. Fourth, although internal validity of logistic regression models in this study may be relatively high, these results may not be as applicable externally. Further studies will be needed to examine a test set of additional massive rotator cuff tears. Finally, the present study was a cross-sectional study of patients with massive rotator cuff tears. The duration of symptoms was not the same among the groups. Although the duration of symptoms had no impact on the radiographic changes of the shoulder, their onset periods did not always correspond with the occurrence of rotator cuff tears; thus, the duration could possibly be inaccurate. Additionally, the causal association between the radiographic findings and each of the factors identified as being associated with radiographic change in this study remains unclear, due to the nature of a cross-sectional study. For example, this study suggested an association between LHB tendon rupture and superior migration of the humeral head, but whether this is a cause or a consequence of superior migration of the humeral head remains unsolved, as has been discussed in previous literatures^4,5,13,21,24–26^. Further longitudinal observational studies will be needed to clarify the progression of the radiographic changes in cases with massive rotator cuff tears.

In conclusion, this study indicated rupture of the infraspinatus and biceps long head tendon as risk factors of superior migration of the humeral head and that rupture of the subscapularis and biceps long head tendon and female sex are risk factors of cuff tear arthropathy.

## Data Availability

The datasets analysed during the current study are available from the corresponding author on reasonable request.
